# Identifying the Impact of Landscape Pattern on Ecosystem Services in the Middle Reaches of the Yangtze River Urban Agglomerations, China

**DOI:** 10.3390/ijerph17145063

**Published:** 2020-07-14

**Authors:** Luwen Liu, Xingrong Chen, Wanxu Chen, Xinyue Ye

**Affiliations:** 1 School of Mathematics and Physics, China University of Geosciences, Wuhan 430074, China; cugliuluwen@126.com (L.L.); chenxingrong2008@163.com (X.C.); 2 Department of Geography, School of Geography and Information Engineering, China University of Geosciences, Wuhan 430074, China; 3 Research Center of Spatial Planning and Human-Environmental System Simulation, China University of Geosciences, Wuhan 430074, China; 4 Department of Landscape Architecture and Urban Planning, Texas A&M University, College Station, TX 77840, USA

**Keywords:** ecosystem services value, landscape pattern metrics, spatial regression analysis, middle reaches of the Yangtze River Urban Agglomerations, China

## Abstract

Clarifying the impact mechanisms of landscape patterns on ecosystem services is highly important for effective ecosystem protection, policymaking, and landscape planning. However, previous literature lacks knowledge about the impact mechanisms of landscape patterns on ecosystem services from a spatial perspective. Thus, this study measured landscape patterns and the ecosystem services value (ESV) using a series of landscape pattern metrics and an improved benefit transfer method based on land-use data from 2015. It explores the impact mechanisms of the landscape pattern metrics on the ESV using the ordinary least-squares method and spatial regression models in the middle reaches of the Yangtze River Urban Agglomerations (MRYRUA), China. We found that forestland was the main landscape type in the MRYRUA, followed by cultivated land, and the fragmentation degree of cultivated land was significantly higher than that of forestland. The findings demonstrate that landscape pattern metrics had a significant impact on ecosystem services, but could vary greatly. Moreover, ecosystem services in the MRYRUA exhibited significant spatial spillover effects and cross-regional collaborative governance was an effective means of landscape planning. This paper acts as a scientific reference and effective guidance for landscape planning and regional ecosystem conservation in MRYRUA and other similarly fast-growing urban agglomerations.

## 1. Introduction

Land-use/land-cover change (LULCC), an important component and determinant of global environmental change, is the main manifestation of landscape pattern changes in the earth’s surface system [1,2]. The worldwide population explosion and rapid advancement of urbanization and industrialization have exacerbated the evolution of landscape patterns and are causing severe interference to the global ecosystem [3,4,5]. Identifying the impact of landscape patterns on ecosystem services is highly significant for ecosystem protection policy-making and landscape planning, especially in fast-growing urban agglomerations in developing countries. At present, the development of new urbanization in China is entering an important stage, with urban agglomerations as the main form [6]. The rapid urbanization in the urban agglomerations has accelerated the evolution of landscape patterns, which has caused a series of severe socioeconomic and ecological issues, such as water shortages [7], food security problems [8], biodiversity degradation, and ecosystem deterioration [9,10], and has seriously threatened the sustainable development of urban agglomerations [11]. However, because of the previous lack of research on the transmission and feedback mechanism of the impact of landscape patterns on ecosystem services from spatial aspects, and the spatial associations and impact mechanism existing between landscape patterns and ecosystem services, these topics need further exploration and analysis. In this context, clarifying the impact mechanism of landscape patterns on ecosystem services has become a focus of decision-makers and scholars.

Landscapes are mosaics of different land-use patterns or heterogeneous regions composed of multiple ecosystems [12]. Essentially, these different ecosystems can often be represented by different land-use or land-cover types. Therefore, a landscape pattern mainly refers to the shape, proportion, and spatial arrangement that constitute ecosystems or land-use or land-cover types [13]. Changes in landscape patterns impact the supply capacity of ecosystem services by causing changes in ecosystem components, structures, ecological processes, and biodiversity [14]. For example, ecosystem services provided by agricultural landscapes generally have a stronger capacity to provide supply services, but have a weaker capacity for regulation services, cultural services, and support services; in contrast, the regulation and support services of forest landscapes are higher, but the capacity for supply services is lower. Different landscape patterns correspond to corresponding ecological processes and have different impacts on ecosystem services [10,15]. Specifically, changes in landscape patterns influence the structure and function of the ecosystem by affecting the types, areas, and spatial distribution of various ecosystems, resulting in the change in material, energy, and ecological flows in the landscape, and ultimately affecting the supply and maintenance of ecosystem services [13,15].

The process of landscape pattern change is the process of a material cycle, energy flow, and ecological flow between human systems and environmental systems. During this process, there is a significant interference of soil, climate, hydrology, and other geochemical cycles, as well as natural factors, such as biodiversity, thus changing the structure, composition, and function of ecosystems [16]. In addition, ecological processes, such as nutrient cycling, soil erosion, and microbial degradation, are spatially and temporally different, which has a huge impact on the supply and maintenance of ecosystem services. Generally speaking, in natural ecosystems with fewer human activities, there are relatively low levels of supply services, but there are relatively high levels of regulation services and support services. In the case of moderate human development, the level of supply services tends to rise rapidly, while the level of regulation services and support services declines. Excessive human disturbances to the ecosystem will cause the degradation of various ecosystem services. In areas with strong human activities, the natural landscape is greatly transformed and the landscape pattern index can reflect the impact of human activities on the landscape structure to a certain extent [17]. Studies on the ecological process of regional landscape change can better explain landscape pattern changes and ecological effects. 

The concept of ecosystem services, which link the ecosystem with human development systems, provides a new perspective for studying the interaction between ecosystems and human systems. The measurement of ecosystem services mainly includes the monetary value, material, and energy methods [10,18]. The benefit transfer method realizes the monetization of ecosystem services based on LULCC data, which can reflect the scarcity and importance of ecosystem services [19], and has been widely used in previous studies because of its feasibility and and the availability of data. The benefit transfer method uses existing valuation studies to infer monetary values from one or more existing study sites for application to target study sites that lack original valuation data [20]. In previous studies, extensive research has been conducted on evaluations, trade-offs, supply and demand, scenario prediction, influence factors, and the optimal regulation of ecosystem services, but there are still inadequacies [9,21,22,23]. The spatialization of the ESV has always been controversial. Previous studies have corrected ESV assessments based on the assumption that ecosystem services’ intensity is linearly associated with biomass [24,25]; however, biomass is not entirely positively correlated with the ESV. For example, aquatic ecosystems contain very little biomass but it plays an important role in hydrological regulation and waste treatment. The relationship between landscape patterns and ecological processes is very complex, often including non-linear and complex coupling with feedback; however, previous literature included analyses of the impact of landscape changes on ecosystem services, the relevant research on the impact of landscape pattern changes on ecosystem services is still insufficient [26,27]. For example, the impact of landscape patterns on various functions provided by ecosystems (e.g., supply services, regulation services, support services, and cultural services) is still unclear [27,28]. 

In addition, previous studies on how landscape pattern changes drive the ESV have neglected ecosystem services as public services that exhibit a strong spatial spillover effect [9]. That is, ecosystem services in a local unit are affected not only by the individual ecosystem services supply capacity but also by the ecosystem services supply capacity of the adjacent unit [29]. In addition, previous research has failed to sufficiently consider the spatial autocorrelation between the ESV and landscape pattern metrics. There is always a strong spatial autocorrelation between the ESV and landscape pattern metrics, such that ignoring the comprehensive consideration of the spatial dependence effect will reduce the ability to explain their relationship [30,31]. Finally, previous research on ESV tends to focus on single units without studying urban agglomerations, whereas examining urban agglomerations can help to promote cross-regional joint governance [32,33]. Therefore, studying the impact of landscape patterns on the ESV from a spatial perspective can adequately grasp the spatial interaction between landscape patterns and ecosystem services and promote the sustainable development of urban agglomerations.

This study took landscape patterns, which are considered to be an important bridge between the landscape and ecosystem, as the breakthrough point and analyzed the impact of landscape patterns on ecosystem services. The findings help to promote the understanding and interpretation of the relationship between landscape patterns and ecosystem services and provide a scientific reference for sustainable development opinions and technical solutions. Consequently, this study selected the MRYRUA as a study case to explore the spatial relationship between landscape patterns and the ESV in the MRYRUA. As a new economic development and a pilot zone for a new type of urbanization in the central and western regions of China, the landscape patterns and the supply capacity of the ecosystem in the MRYRUA have changed significantly. Exploring the impact mechanisms of landscape patterns on the ESV plays an important role in providing effective ecosystem protection policies and relevant landscape planning suggestions. The main parts of this study are as follows: (1) the spatial distribution features of the ESV in the MRYRUA were evaluated with a revised, improved benefit transfer method based on LULCC data from 2015; (2) the spatial distribution features of a series of landscape pattern indexes of the MRYRUA were measured using Fragstats v4.2.1 software (Oregon State University, Corvallis, OR, USA); and (3) the mechanisms of the landscape pattern that drives the ESV were analyzed for the MRYRUA using the least-squares method and spatial regression models, providing scientific support for ecosystem protection and sustainable land use in the MRYRUA. We organized the remainder of this paper as follows. The study area, data sources, and methods are introduced in Section 2; Section 3 introduces the findings of this study; Section 4 presents the discussion and policy implications; and Section 5 presents the conclusions.

## 2. Materials and Methods

### 2.1. Study Area

**Figure 1:**
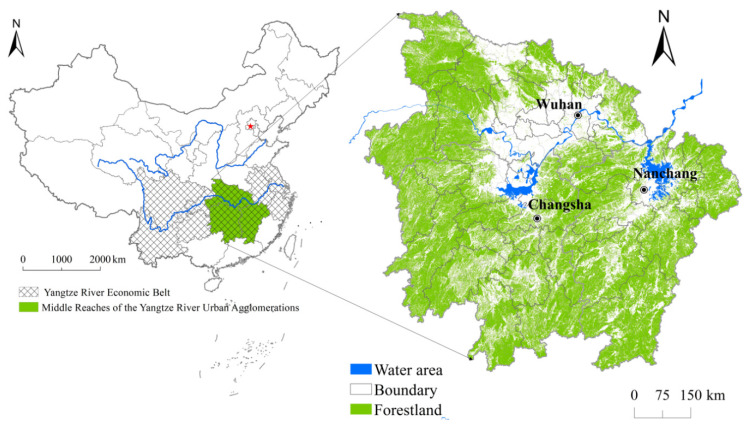
Location of the middle reaches of the Yangtze River Urban Agglomerations (MRYRUA) in China.

The MRYRUA includes the Hunan, Hubei, and Jiangxi Provinces (108°21′–118°28′ E, 24°29′–33°20′ N), and covering 325 counties, and is an important component of national strategies, such as the Yangtze River Economic Belt and the Rise of Central China (as illustrated in Figure 1). The terrain conditions are very complex because the MRYRUA is located in the transition zone from the second to the third step. The MRYRUA is surrounded by Wuling Mountain, Wushan Mountain, Xuefeng Mountain, Nanling Mountain, and Dabie Mountain. The Luoxiao mountain range is located at the junction of Hunan Province and Jiangxi Province, with Jianghan Plain, Dongting Lake Plain, and Poyang Lake Plain distributed along the Yangtze River. The Yangtze River system crosses the study area. The MRYRUA has excellent natural geographical conditions, linking the east with the west and connecting southern China with northern China with convenient transportation. Many major railways in China run through the MRYRUA (e.g., Beijing–Guangzhou Railway and Beijing–Kowloon Railway) and several air routes constitute a comprehensive three-dimensional traffic corridor. The super large urban agglomerations formed by Wuhan Metropolis, Changsha–Zhuzhou–Xiangtan urban agglomerations, and Poyang Lake urban agglomerations have strongly supported the economic development of the MRYRUA and formed a new growth area for China’s economic development. The proportion of forestland in the MRYRUA is very high and has a strong hydrologic regulatory function. In addition, the complex geological structures and rich geomorphic types in the MRYRUA lay a foundation for breeding various organisms. Therefore, the MRYRUA has rich biodiversity resources, plays an important role in maintaining biodiversity, and has several biodiversity regions with international significance, such as the Jianghan Plain wetlands and Dongting Lake Plain wetlands. Therefore, the MRYRUA was selected as the study area to examine the driving mechanisms of the ecosystem services to provide scientific guidance for land-use management and the formulation of reasonable ecological management policies.

### 2.2. Data Sources

The 30 m resolution land-use data and normalized difference vegetation index (NDVI) data with a 1 km resolution from 2015 used in the study were sourced from the Data Center for Resources and Environmental Sciences of the Chinese Academy of Sciences (http://www.resdc.cn) [34,35]. Liu et al. [34] reconstructed China’s LULCC datasets with 5-year intervals from the late 1970s to 2018 using Landsat TM/ETM remote sensing images as the main data source, which has a spatial resolution of 30 × 30 m [34]. Land-use data from 2015 were generated through the human–computer interactive interpretation method based on Landsat 8 remote sensing images (e.g., Landsat 8 operational land imager and GF-2), with reference to 2010 LULCC data. In line with the existing research related to ecosystem services, the land-use data were divided into seven first-class land-use types, namely, cultivated land, forestland, grassland, water area, wetland, construction land, and unused land. Based on field trips, the comprehensive evaluation accuracy of the first-class land-use data was higher than 93% [34]. The grain output data involved in this study were from the *Hubei Statistical Yearbook*, *Hunan Statistical Yearbook*, and *Jiangxi Statistical Yearbook* in 2016, and the grain price data were sourced from the *2016 China Yearbook of Agricultural Price Survey*.

### 2.3. Methodology

#### 2.3.1. Ecosystem Services Value Measurement

The theoretical framework of ESV measurement put forward by Costanza et al. provided a new path for quantifying ecosystem services, and subsequently, extensive research on the ESV has been carried out worldwide using their method [22,36,37,38]. According to Costanza et al. and the expert knowledge of ≥700 ecologists, Xie et al. reclassified ecosystem services and the revised equivalent table for the ESV in China [22,39]. Specifically, the classification of ecosystem services was revised into four primary categories (supply services, regulation services, support services, and cultural services) and nine secondary categories from 17 ecosystem services proposed by Costanza et al. [22,39]. Xie et al. put forward the concept of equivalent value per unit area as a modification of the equivalent table of the ESV [39]. The equivalent value per unit area was defined as the relative importance of various ecosystem services to the grain production of cultivated land, where the equivalent value per unit area of grain production of cultivated land was set to 1. Therefore, we could obtain the equivalent value per unit area of other ecosystem services by comparing their relative importance to the grain production of cultivated land [10,21]. The equivalent value per unit area proposed by Xie et al. was revised using expert knowledge and with reference to the actual situation of China’s ecosystem, but it cannot be used in MRYRUA directly because of the spatial heterogeneity of the ESV. Xie et al. asserted that the ESV has a significant linear relationship with biomass and can be spatialized based on biomass [23,24,39]. However, ecosystem services and biomass do not necessarily have a completely linear relationship. For example, there is a small amount of biomass but a large number of ecosystem services in water areas and wetlands. In the models in previous research, deviations may occur in the revision of the ESV based on the regional biomass. As the equivalent table proposed by Xie et al. uses the economic value of cultivated land per unit area as a reference, Chen et al. thought that it was more reasonable to make a spatial correction of ecosystem services based on the biomass provided by cultivated land [10]. Chen et al. measured the ESV of the MRYRUA based on the grain output and grain price [40]. Using Chen et al.’s research, this study corrects the ESV of the MRYRUA based on the biomass provided by cultivated land [10,40]. The specific equations are as follows:

(1) $$ESV_{k}=\frac{VCI_{k}}{\bar{VCI}}\times \sum_{j=1}^{m}\sum_{i=1}^{n}\left(LA_{i}\times VC_{i}\right)$$

(2) $$AESV_{k}=\frac{ESV_{k}}{\sum_{i=1}^{n}LA_{i}}$$

where *ESV_k_* refers to the corrected ESV of the *k*th county (RMB), *LA_i_* represents the area of land-use type *i* (hm^2^), *VC_i_* is the equivalent coefficient of the ESV (RMB/(hm^2^·a)), *VIC_k_* is the biomass on the cultivated land of the *i*th county unit, and $\bar{VCI}$ is the average of the biomass on the cultivated land in the MRYRUA. *AESV_k_* is the average ecosystem services value of the *k*th county (RMB/hm^2^), which is calculated by dividing the corrected ESV of the *k*th county by the corresponding county unit area.

#### 2.3.2. Landscape Pattern Index

The landscape pattern index can effectively reflect the spatial allocation and structural characteristics of landscape patterns [17]. To reveal the landscape pattern features in the MRYRUA, based on previous studies [26,41,42], this study selected a series of landscape pattern metrics from patch level and landscape level, including the number of patches (NP), patch density (PD), patch edge density (ED), percentage of landscape (PLAND), landscape shape index (LSI), Shannon diversity index (SHDI), Simpson diversity index (SIDI), interspersion juxtaposition index (IJI), landscape division index (DIVISION), patch cohesion index (COHESION), splitting index (SPLIT), area-weighted mean patch area (AREA_AM), area-weighted mean patch shape index (SHAPE_AM), area-weighted mean patch fractal index (FRAC_AM), aggregation index (AI), and contagion index (CONTAG). Moreover, the landscape pattern features in the MRYRUA were measured at the level of class level and the landscape level, where the specific equation was taken from McGarigal et al. [17]. In this study, the software of Fragstats v4.2.1 software was used to calculate the landscape pattern index. 

#### 2.3.3. Spatial Autocorrelation Test

To explore the spatial agglomeration and dispersion characteristics between the average ESV and landscape pattern indexes, this study adopted the bivariate global spatial autocorrelation method to measure the spatial relationship between them to determine the model selection [43]. The global bivariate Moran’s *I* can be used to study whether there is a spatial correlation between the average ESV and landscape pattern indexes of the MRYRUA [44]. The equation is as follows:

(3) $$I=\frac{N\sum_{i}^{N}\sum_{j\neq i}^{N}W_{ij}z_{i}^{e}z_{j}^{u}}{(N-1)\sum_{i}^{N}\sum_{j\neq i}^{N}W_{ij}}$$

where *I* is the global bivariate spatial autocorrelation index, *N* is the number of research units, *W_ij_* is the adjacent spatial weight matrix, *z_i_^e^* is the ESV of the *i*th unit, and *z_j_^u^* is the landscape pattern index of the *j*th unit. The global Moran’s *I* value is generally between –1 and 1. If the value is greater than zero, it indicates that there is a positive autocorrelation; if the value is less than zero, it indicates that there is a negative autocorrelation; and if the value is close to zero, it indicates that there is a random distribution. Additionally, the *p*-value is often used as a significance test.

#### 2.3.4. Spatial Regression Analysis

Four regression models were used in this study to verify the impact of the landscape pattern index on ecosystem services, including the ordinary least-squares method, spatial lag model, spatial error model, and spatial error model with lag dependence. The specific methods are as follows.

Ordinary least-squares method (OLS): The standard linear regression model assumes that the random error term of the model is independent with a normal distribution and confirming whether the model hypothesis is satisfied can be determined through a model diagnosis. The OLS model comprehensively considers the importance of independent variables to dependent variables without regard to the influence of the neighborhood [27]. The equation is as follows:

(4) $$AESV_{t}=X_{t}\beta +\varepsilon$$

Spatial lag model (SLM): The SLM model assumes that spatial autocorrelation occurs with the dependent variables, emphasizes the neighborhood effect, and considers the spatial diffusion (spillover effect) of the dependent variables among geographic units [27]. The equation is as follows:

(5) $$AESV_{t}=X_{t}\beta +\rho W_{1}AESV_{t}+\varepsilon$$

Spatial error model (SEM): The spatial dependence of SEM exists in the disturbance error term. The model measures the impact of the error shock of the dependent variables of adjacent geographical units on the observed values in this area [27]. The equation is as follows:

(6) $$AESV_{t}=X_{t}\beta +\varepsilon ,\varepsilon =\lambda W_{2}\varepsilon +\xi$$

Spatial error model with lag dependence (SEMLD). The spatial lag model and spatial error model are an oversimplification, which may exclude other possible spatial autocorrelation mechanisms, such as the simultaneous existence of a spatial lag and error autocorrelation [27,45,46]. The SEMLD model includes the spatial lag model and the spatial error model, which is a spatial autoregressive model enhanced by adding spatial lag dependent variables. The equation can be expressed as a combination of Equations (5) and (6), as follows:

(7) $$AESV_{t}=X_{t}\beta +\rho W_{1}AESV_{t}+\varepsilon ,\varepsilon =\lambda W_{2}\varepsilon +\xi$$

where *AESV_t_* is the matrix of the average ESV in year *t*; *X_t_* is the *n* × *k* independent variable matrix in year *t*, where *n* is the number of research units and *k* is the number of explanatory variables; *β* is the coefficient vector of *X_t_*, which indicates the influence of independent variables on the dependent variables; *ρ* is the spatial lag parameter, λ is the spatial error parameter, *ε* is the vector of the random error term, and *W*_1_ and *W*_2_ are the spatial weight matrixes of the lag terms and error terms, respectively. 

## 3. Results and Analysis

### 3.1. Ecosystem Services Value in the MRYRUA

Using Equations (1) and (2), the ESV supply capacity of the MRYRUA was measured for 2015; the ESV provided by the ecosystems in the MRYRUA in 2015 was RMB 2770.351 billion. Compared with the research results of Chen et al. (2019; RMB 2611.560 billion) and Yang et al. (2017; RMB 2758.851 billion), our result was greater but the difference is not significant [40,47]. Compared with the research results of Liu et al., the ESV of the MRYRUA in 2010 was only RMB 877.09 billion [48]. Specifically, supply services, regulation services, support services, and cultural services values of the MRYRUA in 2015 were RMB 308.754 billion, RMB 1485.153 billion, RMB 781.831 billion, and RMB 194.612 million, respectively. Regulation services were significantly higher than the other types of services and the value of cultural services was the lowest. 

**Figure 2:**
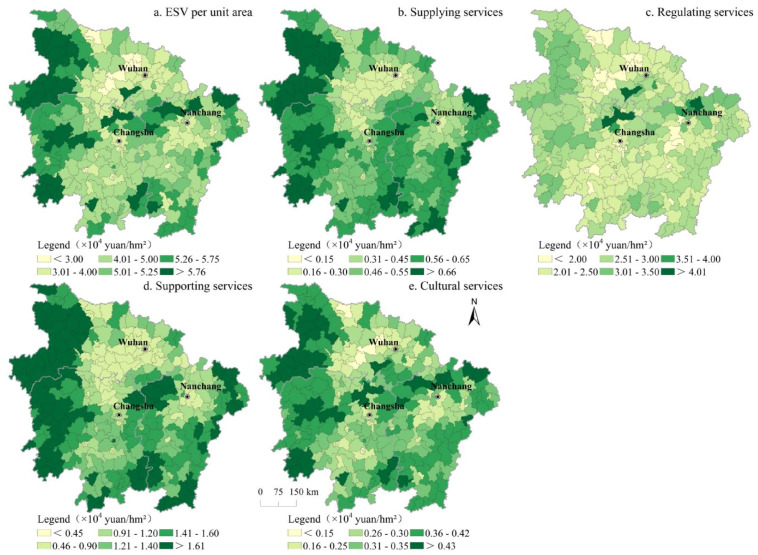
Spatial distribution of the average ecosystem services value (ESV) of the MRYRUA in 2015.

The spatial distribution patterns of the average ESV, average supply services, average regulation services, average support services, and average cultural services are provided in Figure 2. The counties with a low average ESV of the MRYRUA were mainly distributed in Jianghan Plain, Poyang Lake Plain, and Dongting Lake Plain, especially in large cities and surrounding counties and districts, as well as counties and districts along important traffic routes. The areas with a high average ESV were mainly distributed in Wu Mountain in the west of the MRYRUA and Dabie Mountain north of Hubei Province, Xuefeng Mountain in the west and central Hunan Province, the Nanling Mountains in the south, Wuyi Mountain in east Jiangxi Province, and Luoxiao Mountain between Jiangxi Province and Hunan Province. Comparing the different ecosystem service types, the spatial distribution patterns of supply services, regulation services, support services, and cultural services were similar. Moreover, the regulating capacity of ecosystems in Dongting Lake and Poyang Lake areas was significantly higher than those in other areas, which was mainly due to the strong waste treatment capacity of the water body. In addition, the supply capacity of ecosystem services in counties along the Yangtze River was significantly higher than those in other regions. Because of the large differences in natural conditions and the socioeconomic development levels of mountain and plain areas, the ESV provided by the ecosystems also exhibited great differences.

### 3.2. Landscape Pattern Indexes in the MRYRUA

Forestland and cultivated land were the main landscape types in the MRYRUA, accounting for 58.06% and 30.14% of the total coverage, respectively (as illustrated in Table 1). However, the number of patches of cultivated land was significantly higher than those of forestland, indicating that the fragmentation of cultivated land was more serious. Construction land accounted for only 3.09% but the number of patches was second only to that of cultivated land, indicating that the fragmentation of construction land was also severe. The patch density of cultivated land and construction land was 0.31 and 0.18, respectively, which was significantly higher than those of other landscape types. In terms of edge density, the edge density of cultivated land and forestland was 21.80 and 20.17, respectively, which were significantly higher than those of other landscape types. We can find similar features for the area-weighted mean patch area, the area-weighted mean patch fractal index, the area-weighted mean patch shape index, and the percentage of landscape in all landscapes. These landscape pattern indexes were the largest for forestland, and those of unused land were the lowest among all the landscapes. The interspersion juxtaposition index of the wetland landscape was the largest (67.50), followed by that of water area (65.89), while that of forestland was the smallest (39.21). The cohesion degree of construction land was the lowest (92.98), and that of forestland was the highest (99.96) among all the landscapes. The landscape splitting index of unused land was the highest, followed by that of construction land, while that of forestland was the lowest. In contrast, the aggregation index of forestland was the highest (92.15), while that of unused land was the lowest (70.19). In terms of the spatial distribution of various landscape pattern indexes, the patch density in the central areas of key cities was relatively large and similar distribution characteristics were found in the interspersion juxtaposition index. The landscape shape index in the central areas of core cities was significantly lower than that found in other counties. Similar distribution characteristics were found in the patch cohesion index and aggregation index. The Shannon diversity index, landscape division index, and splitting index in the plain areas were higher than those in the surrounding mountain areas.

### 3.3. Spatial Regression Analysis

#### 3.3.1. Specification of Variables

**Figure 3:**
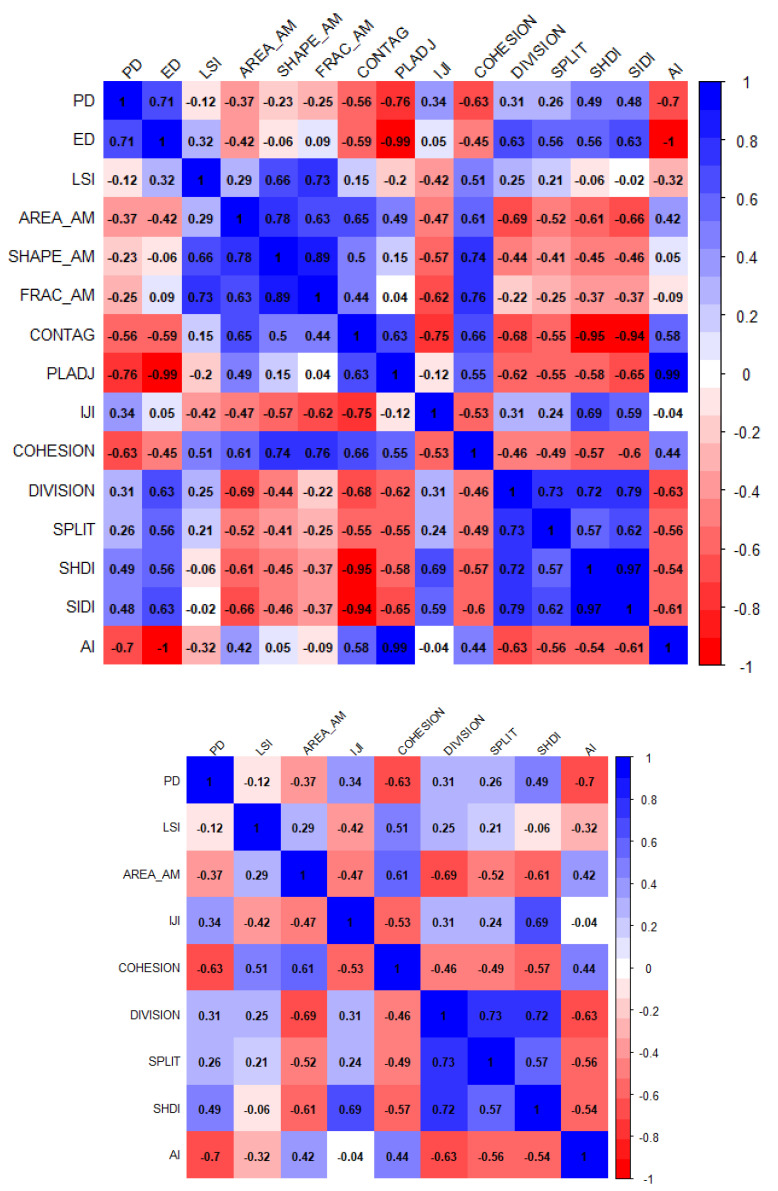
Correlation matrixes of the landscape pattern metrics. Note: The correlation matrix of landscape pattern metrics in model 1 is is shown above and the correlation matrix of the landscape pattern metrics in model 2 is shown below.

In this study, several factors were included in the models as the dependent variables for the 325 county units of the MRYRUA in 2015, such as the average ESV, average supply services, average regulation services, average support services, and average cultural services. The independent variables of the models were the landscape pattern indexes calculated in Section 2.3.2. Multiple landscape pattern indexes may lead to the existence of multicollinearity, where the multicollinearity diagnosis for landscape pattern indexes was conducted using Stata 15.0 SE version software (StataCorp, College Station, TX, USA). It is generally believed that the larger the value of the variance inflation factor (VIF), the stronger the collinearity. In this study, nine factors with a VIF less than eight were selected as independent variables of the models (Table 2). To further prove the rationality of the selected landscape pattern metrics, the correlation matrixes of all the landscape pattern metrics and the selected landscape pattern metrics were provided in this study (Figure 3). The correlation between the selected landscape pattern indexes was demonstrated as being significantly reduced.

#### 3.3.2. Bivariate Spatial Correlation Test between the Ecosystem Services Value and Landscape Pattern Metrics in the MRYRUA

The bivariate spatial autocorrelation of the average ESV, as well as the average supply services, average regulation services, average support services, and average cultural services and the landscape pattern index were tested with the help of Geoda095i software (University of Chicago, Chicago, IL, USA) by using the first-order queen continuity spatial matrix of MRYRUA from 2015 (as illustrated in Table 3). The bivariate spatial autocorrelation index between the ESV and the aggregation index was negative and significant at the level of 0.05 level. The bivariate spatial autocorrelation index between the average regulation services and average cultural services and the aggregation index was positive and also significant at the level of 0.05 level. The bivariate spatial autocorrelation index between the average support services and aggregation index was positive but not significant. The bivariate spatial autocorrelation indexes between other types of ecosystem services and landscape pattern indexes were all significant at the level of 0.0001 level. Specifically, the bivariate spatial autocorrelation index between the average ESV, as well as average supply services, average regulation services, average support services, and average cultural services and the PD, IJI, DIVISION, SPLIT, and SHDI were negative, while the bivariate spatial autocorrelation index between the average ESV, as well as the average supply services, average regulation services, average support services, and average cultural services and the LSI, AREA_AM, and COHESION were positive. Based on the results of the bivariate spatial autocorrelation analysis between the ESV and landscape pattern index, there was a significant spatial dependence between the ecosystem services and the landscape pattern index. Therefore, it was necessary to fully consider the spatial dependence in the analysis of the impact of the landscape pattern index on ecosystem services in the MRYRUA.

#### 3.3.3. Impact of the Landscape Pattern on Ecosystem Services in the MRYRUA

The results of the bivariate spatial autocorrelation analysis demonstrated that there was a significant spatial dependence between the ESV and the landscape pattern index for the MRYRUA. According to the OLS regression results, the residual test results passed the significance test at the level of 0.0001 level, which demonstrated that there was significant spatial autocorrelation in the residuals. If the traditional regression model was adopted, the model hypothesis was violated. Therefore, based on the results of the residual test and spatial autocorrelation test, the spatial regression model needed to be considered in the exploration of the relationship between the ESV and the landscape pattern index (as illustrated in Table 4). The spatial regression model considered the spatial effect, which could overcome the problem of setting the deviation of a traditional econometric model to a certain extent. In this study, a set of regression models for cross-section data were adopted, including the spatial lag model (SLM), spatial error model (SEM), and spatial error model with lag dependence (SEMLD), to explore the impact mechanism of the landscape pattern index on the ecosystem services. The items of the robust LM (lag), robust LM (error), and LM (lag and error) were significant at the level of 0.0001 level in all the models, indicating that the SEM, SLM, and SEMLD models could be employed in this study. To select the model with the best performance, this study listed the regression results of three spatial regression models (as illustrated in Table 5) and compared the log-likelihood (LogL), Akaike information criterion (AIC), and Schwartz’s Bayesian information criterion (SC) of these three spatial models to determine the suitability of the models. The larger the LogL, and the smaller the AIC and the SC, the better the fitting degree. The fitting degree of the spatial regression models was better than those of the OLS model, while the LogL value of the SEMLD model was the largest of the four models, and its AIC and SC were the smallest of the four models. Therefore, the SEMLD model was the most appropriate choice in this study. In all of the models, the statistical values of the Breusch–Pagan test and Koenker–Bassett test passed the significance test at the level of 0.0001 level, indicating that there was no heteroscedasticity in the independent variables.

The regression results demonstrated that the spatial relationship between the patch density (PD) and the average ESV, average supply services, average regulation services, average support services, and average cultural services were negative, indicating that the patch density had a negative impact on ecosystem services. In the SEMLD model, a 1% increase in patch density would lead to a decrease of 0.138%, 0.195%, 0.093%, 0.201%, and 0.157% in the average ESV, average supply services, average regulation services, average support services, and average cultural services, respectively. The aggravation of landscape fragmentation would affect the composition, structure, ecological process, and biodiversity of the ecosystem, and reduce the supply capacity of ecosystem services. The same results were found by Su et al. and Yushanjiang et al. [41,42]. The regression coefficient of the landscape shape index (LSI) was negative but was significant only in a few models. The impact of the area-weighted mean patch area (AREA_AM) on the ecosystem services was positive and significant in all models. The interspersion juxtaposition index (IJI) was not significant in the spatial lag model but had a significant negative association with the ecosystem services in other models. The results demonstrated that the larger the adjacent edge length between the different patches, the weaker the ecosystem services’ supply capacity. There was a significant positive relationship between the patch cohesion index (COHESION) and ecosystem services, indicating that the stronger the landscape connectivity, the stronger the ecosystem services’ supply capacity. The regression coefficients of the landscape division index (DIVISION) were all positive but only significant in only a few models. The regression coefficient of the splitting index (SPLIT) was also positive and not significant in all models. The Shannon diversity index (SHDI) had different impacts on the average ESV, average supply services, average regulation services, average support services, and average cultural services. Specifically, the increase of the Shannon diversity index could promote the improvement of the average ESV, average regulation services, and average cultural services. In contrast, the increase of the Shannon diversity index could lead to the degradation of the average supply services and average support services. The spatial relationship between the aggregation index (AI) and the average ESV, average supply services, average support services, and average cultural services were negative and significant, while the impact of the AI on the average regulation services was not significant. The spatial lag terms in all the spatial regression models were significant at the level of 0.0001 level, and the coefficient was positive, indicating that the improvement of ecosystem services in the surrounding units would lead to the improvement of ecosystem services in the local unit. In addition, the spatial error term in the spatial model was significant in all models, indicating that other factors affected the ecosystem services besides the landscape pattern index.

## 4. Discussion and Policy Implications

### 4.1. Impact of the Landscape Pattern on the Ecosystem Services Value

Based on relevant theories and methods of landscape ecology and ecosystem services, this research explored the spatial relationship between the landscape pattern and ecosystem services value in the MRYRUA. The change in landscape pattern had an important impact on the structure, function, and the process of the regional ecosystem. There was a significant negative association between ecosystem services and PD, LSI, IJI, and AI, while there was a significant positive association between AREA_AM, COHESION, DIVISION, SPLIT, and the ecosystem services. The increase in the Shannon diversity index could promote the improvement of the average ESV, average regulation services, and average cultural services, while the increase of the Shannon diversity index could lead to the degradation of the supply and support services in the MRYRUA. The results demonstrated that the landscape pattern had an important impact on the components, structure, function, and biochemical process of regional ecosystem, and eventually led to a change in the ecosystem services [15,28,29]. The landscape pattern index could demonstrate the changes in the intensity of human activities more effectively. Although the study mainly explored the impact of landscape patterns on the ecosystem services value without considering other driving factors in the MRYRUA, studying the impact of landscape patterns on the ecosystem services is the basis for a deep understanding of the relationship between human activities and landscape pattern evolution [15,28,29]. The evolution of a landscape pattern is not only related to natural factors but is also closely related to human factors [49]. With the rapid development of urbanization and the economy, as well as improvements in the transportation infrastructure, human activities in urban agglomerations have become the main reason for the evolution of landscape patterns, and have changed the original composition and structure of the ecosystem, as well as the sustainable supply of ecosystem services.

Land use will cause landscape diversity changes in terms of its spatial structure, functional mechanism, and temporal dynamics, which will transmit to the ecosystem through ecological processes, which will have an impact on the biodiversity of the ecosystem, which is composed of heredity, species, and ecosystem diversity. However, biodiversity is positively correlated with ecosystem stability [50], and the stability of ecosystem services is therefore affected. Landscape patterns can drive changes in ecosystem services, and ecosystem services, in turn, will provide feedback to the land-use system. Specifically, different land-use patterns correspond to different landscape structures and landscape spatial configurations, which leads to changes in characteristics of ecosystem services, such as their heterogeneity, stability, and diversity, resulting in changes in the value of ecosystem services in the region. Conversely, ecosystems impact land-use patterns by providing biodiversity and ecosystem services and result in negative environmental, economic, and social implications. Subsequently, the corresponding measures and means, such as land-use engineering, land-use landscape planning, and land-use-related policies, can be developed, leading to positive changes in the land-use system.

### 4.2. Policy Implications

The results of this study demonstrated that the spatial regression model could better explain the spatial relationship between the landscape pattern index and ecosystem services. The quantitative analysis of the relationship between the landscape pattern index and ecosystem services can help to better understand how changes in landscape patterns affect ecosystem services [41]. In addition, the analysis of the relationship between the landscape pattern index and ecosystem services can guide land-use planning and improve ecosystem protection policymaking. The MRYRUA is an important part of the Yangtze River Economic Belt and Rise of Central China strategies. Based on the analysis results, this study puts forward the following suggestions. Land-use change was the main cause of landscape pattern changes, and rapid urbanization had a severe impact on the landscape patterns and ecosystem services. In the process of ecosystem protection, it is necessary to consider the landscape pattern changes caused by land use, improve landscape connectivity, and reduce patch fragmentation [41]. In terms of creating policy, the goal of environmental protection should be taken as the constraint condition of socioeconomic development and be incorporated into the performance appraisal system of administrative agencies at all levels to alleviate the overall deterioration trend of the ecosystem from the aspects of technology, intensive land use, land-use planning, environmental protection investment, and relevant policies and systems [51,52]. The ecosystem services in the MRYRUA had a strong spatial spillover effect. The difference in land-use policies and environmental regulations among different counties was the main reason for the spatial spillover of ecological deterioration. The land-use policies and environmental regulations in underdeveloped areas were always weaker than those in more developed areas, which drove high-polluting enterprises to move toward areas with less-restrictive land-use policies and fewer environmental regulations, and led to the spatial spillover of environmental deterioration. The ecosystem deterioration of a county was affected not only by the local economic and social activities but also by neighboring counties or further areas. Therefore, the formulation of environmental protection policies cannot be limited to a single county and cross-regional collaborative governance should be realized [27].

### 4.3. Limitations and Future Directions

Based on the improved benefit transfer method, this study evaluated the ESV, supply services, regulation services, support services, and cultural services in the MRYRUA. However, there were still some limitations in the method and the subjective assumption of the equivalent factor table in the construction was inevitable. In future studies, other models can be adopted to evaluate ecosystem services, such as the Integrated Valuation of Ecosystem Services and Trade-offs model to measure capacities, such as soil and water conservation, water yield, carbon storage, and water purification [53]. Future studies can be aimed to further measure the specific impact of the landscape pattern index on various ecosystem functions (e.g., soil and water conservation capacity, water yield capacity, carbon storage capacity, and water purification) [54]. In addition, this study only explored the impact of landscape patterns on ecosystem services and lacked the comprehensive consideration of other factors. In future studies, we need to comprehensively consider the influencing factors of ecosystem services.

## 5. Conclusions

Based on the LULCC data of the MRYRUA from 2015, this study measured the spatial features of landscape patterns in the MRYRUA and measured the spatial distribution characteristics of the ESV based on the improved benefit transfer method. Spatial regression models were used to analyze the impact mechanism of the landscape pattern index on ecosystem services in the MRYRUA. The findings were as follows: 

(1) Forestland and cultivated land were the main landscape types in the MRYRUA but the fragmentation degree of the cultivated land landscape was significantly higher than that of forestland. The edge density of cultivated land and forestland was significantly higher than that of other landscape types. The interspersion juxtaposition index of wetlands and water areas was the highest, while that of forestland was the smallest. The cohesion degree of construction land was the lowest, while that of forestland was the highest. The landscape splitting index of unused land was the highest, while that of the forestland was the lowest. In contrast, the aggregation index of forestland was the highest, while that of unused land was the lowest.

(2) In 2015, the ESV of the MRYRUA was RMB 2770.351 billion, where the value of regulation services was significantly higher than other service types and the supply capacity of cultural services was the lowest. The supply capacity of ecosystem services in the mountainous areas around the MRYRUA was significantly higher than that in the plain areas, especially in the surrounding areas of big cities, as well as counties along the main traffic routes.

(3) Except for the aggregation index, the bivariate spatial autocorrelation indexes between the landscape pattern indexes and the ecosystem services were all significant at the level of 0.0001 level. The bivariate spatial autocorrelation indexes between the ecosystem services and PD, IJI, DIVISION, SPLIT, and SHDI were negative, while the bivariate spatial autocorrelation indexes between ecosystem services and LSI, AREA_AM, and COHESION were positive.

(4) The regression results demonstrated that there was a significant negative relationship between the ecosystem services and PD, LSI, IJI, and AI, while there was a significant positive association between the ecosystem services and AREA_AM, COHESION, DIVISION, and SPLIT. The increase in the Shannon diversity index could promote the improvement of the average ESV, average regulation services, and average cultural services, while the increase in the Shannon diversity index could lead to the degradation of supply and support services.

## Figures and Tables

**Table 1:** The different landscape indexes of each landscape type in the MRYRUA in 2015.

Land Type	PLAND	NP	PD	ED	LSI	AREA_AM	SHAPE_AM	FRAC_AM	IJI	COHESION	DIVISION	SPLIT	AI
Forestland	58.06	59,679.00	0.11	20.17	500.22	5,559,136.18	145.50	1.38	39.21	99.96	0.94	17.50	92.15
Grassland	3.65	28,955.00	0.05	3.22	318.38	933.42	3.86	1.15	44.59	94.89	1.00	1,659,361.44	80.08
Cultivated land	30.14	176,274.00	0.31	21.80	746.59	472,195.68	50.34	1.32	48.04	99.69	1.00	396.78	83.73
Construction land	3.09	101,248.00	0.18	3.83	408.82	2,522.83	3.91	1.14	45.03	92.98	1.00	723,842.77	72.20
Water area	3.95	34,555.00	0.06	3.00	283.69	266,930.25	33.21	1.26	65.89	99.50	1.00	5,354.71	82.95
Unused land	0.02	586.00	0	0.02	33.49	893.42	5.00	1.14	64.72	93.99	1.00	362,468,475.71	70.19
Wetland	1.10	8,769.00	0.02	0.70	126.46	30,141.46	5.79	1.17	67.50	97.53	1.00	170,671.10	85.64

Notes: PLAND represents percentage of landscape; NP represents number of patches; PD represents patch density; ED represents patch edge density; LSI represents landscape shape index; AREA_AM represents area-weighted mean patch area; SHAPE_AM represents area-weighted mean patch shape index; FRAC_AM represents area-weighted mean patch fractal index; IJI represents interspersion juxtaposition index; COHESION represents patch cohesion index; DIVISION represents landscape division index; SPLIT represents splitting index; AI represents aggregation index.

**Table 2:** The multi-collinearity diagnostics of the regression equation.

Model 1	PD	ED	LSI	AREA_AM	SHAPE_AM	FRAC_AM	CONTAG	PLADJ	IJI	COHESION	DIVISION	SPLIT	SHDI	SIDI	AI
VIF	4.86	758.36	11.89	8.90	19.55	24.04	30.38	474.51	8.71	21.00	13.02	5.20	42.40	48.50	986.40
Model 2	PD	LSI	AREA_AM	IJI	COHESION	DIVISION	SPLIT	SHDI	AI						
VIF	4.35	6.37	3.95	4.02	6.65	6.86	3.09	5.53	6.78						

**Table 3:** Bivariate Moran’s *I* between the average ESV and the landscape pattern metrics for the MRYRUA in 2015.

Ecosystem Services Type	PD	LSI	AREA_AM	IJI	COHESION	DIVISION	SPLIT	SHDI	AI
Ecosystem services	–0.185 ***	0.328 ***	0.387 ***	–0.369 ***	0.373 ***	–0.161 ***	–0.168 ***	–0.293 ***	–0.035 *
Supply services	–0.176 ***	0.370 ***	0.424 ***	–0.505 ***	0.368 ***	–0.194 ***	–0.189 ***	–0.438 ***	–0.003
Regulation services	–0.159 ***	0.236 ***	0.273 ***	–0.184 ***	0.310 ***	–0.090 ***	–0.110 ***	–0.112 ***	0.056 *
Support services	–0.176 ***	0.363 ***	0.433 ***	–0.488 ***	0.368 ***	–0.205 ***	–0.198 ***	–0.424 ***	0.006
Cultural services	–0.177 ***	0.286 ***	0.368 ***	–0.320 ***	0.340 ***	–0.165 ***	–0.166 ***	–0.253 ***	0.049 *

Note: *** *p* ≤ 0.001 and * *p* ≤ 0.05.

**Table 4:** Regression results of the ordinary least-squares (OLS) method.

Variables	Ecosystem Services	Supply Services	Regulation Services	Support Services	Cultural Services
PD	−0.343 ***	−0.367 ***	−0.199 ***	−0.408 ***	−0.392 ***
	(0.088)	(0.090)	(0.071)	(0.093)	(0.096)
LSI	−0.252 **	−0.200 *	−0.237 ***	−0.137	−0.177
	(0.084)	(0.085)	(0.067)	(0.089)	(0.091)
AREA_AM	0.756 ***	0.765 ***	0.527 ***	0.746 ***	0.691 ***
	(0.086)	(0.088)	(0.069)	(0.091)	(0.094)
IJI	−0.212	−0.210 **	−0.149 **	−0.203 **	−0.215 **
	(0.068)	(0.070)	(0.055)	(0.073)	(0.075)
COHESION	0.463 ***	0.331 ***	0.443 ***	0.251 **	0.318 **
	(0.088)	(0.090)	(0.071)	(0.094)	(0.097)
DIVISION	0.294 ***	0.386 ***	0.196 **	0.300 ***	0.179 *
	(0.083)	(0.085)	(0.067)	(0.088)	(0.090)
SPLIT	0.080	0.038	0.099	0.003	0.035
	(0.068)	(0.069	(0.055)	(0.072)	(0.074)
SHDI	0.089	−0.551 ***	0.364 ***	−0.421 ***	0.223 *
	(0.090)	(0.092)	(0.072)	(0.096)	(0.098)
AI	−0.448 ***	−0.747 ***	−0.167 *	−0.689 ***	−0.355 **
	(0.105)	(0.108)	(0.085)	(0.112)	(0.115)
Constant	0.441 ***	0.916 ***	0.024	0.914 ***	0.452 ***
	(0.124)	(0.127)	(0.100)	(0.132)	(0.136)
Moran’s *I* (error)	0.349 ***	0.421 ***	0.311 ***	0.438 ***	0.371 ***
LM (lag)	165.066 ***	216.568 ***	120.282 ***	227.081 ***	163.825 ***
Robust LM (lag)	64.488 ***	79.676 ***	40.185 ***	76.445 ***	49.755 ***
LM (error)	100.916 ***	147.320 ***	80.170 ***	159.537 ***	114.453 ***
Robust LM (error)	0.338	10.428 ***	0.073	8.902 **	0.384
Lagrange multiplier (SARMA)	165.404 ***	226.996 ***	120.355 ***	235.983 ***	164.208 ***
Heteroscedasticity test					
Breusch−Pagan test	87.919 ***	90.855 ***	89.060 ***	80.464 ***	75.053 ***
Koenker−Bassett test	64.657 ***	51.939 ***	49.313 ***	49.842 ***	56.861 ***
Measures of fit					
Log likelihood	233.542	226.648	303.811	213.697	204.816
AIC	−447.083	−433.296	−587.622	−407.395	−389.631
SC	−409.245	−395.458	−549.784	−369.557	−351.793
*R* ^2^	0.606	0.720	0.502	0.680	0.510
*N*	325	325	325	325	325

Note: *** *p* ≤ 0.001, ** *p* ≤ 0.01, and * *p* ≤ 0.05. The standard deviations are in parentheses. LM—Lagrange multiplier, AIC—Akaike information criterion, and SC—Schwartz’s criterion.

**Table 5:** Regression results of the spatial lag model (SLM), spatial error model (SEM), and spatial error model with lag dependence (SEMLD).

Explanatory Variables	Ecosystem Services			Supply Services			Regulation Services			Support Services			Cultural Services		
	SLM	SEM	SEMLD	SLM	SEM	SEMLD	SLM	SEM	SEMLD	SLM	SEM	SEMLD	SLM	SEM	SEMLD
PD	−0.206 **	−0.404 ***	−0.138 *	−0.178 **	−0.362 ***	−0.195 ***	−0.140 *	−0.260 ***	−0.093 *	−0.207 ***	−0.409 ***	−0.201 ***	−0.249 ***	−0.436 ***	−0.157 **
	(0.063)	(0.077)	(0.056)	(0.056)	(0.065)	(0.058)	(0.055)	(0.067)	(0.049)	(0.057)	(0.067)	(0.058)	(0.070)	(0.085)	(0.060)
LSI	−0.112	−0.162 **	−0.077	−0.096	−0.120 *	−0.090	−0.117 *	−0.146 **	−0.075	−0.052	−0.093	−0.044	−0.070	−0.120	−0.036
	(0.059)	(0.063)	(0.056)	(0.052)	(0.052)	(0.053)	(0.053)	(0.056)	(0.049)	(0.054)	(0.054)	(0.054)	(0.066)	(0.069)	(0.060)
AREA_AM	0.353 ***	0.412 ***	0.233 ***	0.284 ***	0.291 ***	0.241 ***	0.296 ***	0.332 ***	0.193 ***	0.266 ***	0.289 ***	0.209 ***	0.334 ***	0.384 ***	0.194 **
	(0.064)	(0.068)	(0.062)	(0.057)	(0.057)	(0.059)	(0.056)	(0.059)	(0.054)	(0.058)	(0.059)	(0.060)	(0.070)	(0.075)	(0.066)
IJI	−0.172 ***	−0.086	−0.178 ***	−0.165 ***	−0.085	−0.143 ***	−0.125 **	−0.062	−0.129 **	−0.159 ***	−0.076	−0.144 ***	−0.173 **	−0.085	−0.179 ***
	(0.048)	(0.053)	(0.045)	(0.043)	(0.044)	(0.043)	(0.043)	(0.047)	(0.040)	(0.044)	(0.045)	(0.044)	(0.054)	(0.059)	(0.049)
COHESION	0.247 ***	0.273 ***	0.191 ***	0.210 ***	0.182 **	0.195 ***	0.244 ***	0.277 ***	0.167 **	0.141 *	0.123	0.125 *	0.145 *	0.172 *	0.094
	(0.063)	(0.076)	(0.058)	(0.056)	(0.063)	(0.058)	(0.057)	(0.066)	(0.053)	(0.057)	(0.066)	(0.058)	(0.070)	(0.083)	(0.062)
DIVISION	0.086	0.138 *	0.029	0.105 *	0.111 *	0.080	0.079	0.114 *	0.035	0.051	0.077	0.022	0.031	0.084	−0.019
	(0.059)	(0.062)	(0.056)	(0.053)	(0.051)	(0.053)	(0.052)	(0.055)	(0.049)	(0.054)	(0.053)	(0.054)	(0.066)	(0.069)	(0.060)
SPLIT	0.099 *	0.083	0.108 *	0.113 **	0.079	0.113 **	0.078	0.074	0.071	0.088 *	0.057	0.095 **	0.060	0.050	0.076
	(0.048)	(0.051)	(0.045)	(0.043)	(0.042)	(0.043)	(0.042)	(0.045)	(0.040)	(0.043)	(0.043)	(0.043)	(0.053)	(0.056)	(0.049)
SHDI	0.155 *	0.058	0.194 ***	−0.186 **	−0.340 ***	−0.206 ***	0.277 ***	0.246 ***	0.242 ***	−0.098	−0.252 ***	−0.085	0.229 **	0.141	0.248 ***
	(0.064)	(0.075)	(0.058)	(0.060)	(0.062)	(0.060)	(0.056)	(0.066)	(0.052)	(0.061)	(0.064)	(0.060)	(0.071)	(0.083)	(0.063)
AI	−0.127 ***	−0.293 ***	−0.021	−0.230 ***	−0.401 ***	−0.221 **	−0.045	−0.128	0.006	−0.183 **	−0.367 ***	−0.140	−0.085	−0.225 *	0.026
	(0.077)	(0.088)	(0.071)	(0.070)	(0.074)	(0.072)	(0.067)	(0.077)	(0.061)	(0.071)	(0.076)	(0.072)	(0.085)	(0.097)	(0.075)
Constant	0.046	0.619 ***	−0.094	0.262 **	0.719 ***	0.268 **	−0.107	0.240 *	−0.169 *	0.258 **	0.772 ***	0.209 *	0.090	0.595 ***	−0.071
	(0.091)	(0.115)	(0.081)	(0.084)	(0.112)	(0.089)	(0.079)	(0.099)	(0.069)	(0.085)	(0.112)	(0.088)	(0.100)	(0.125)	(0.085)
Spatial lag term	0.693 ***		0.894 ***	0.710 ***		0.762 ***	0.639 ***		0.909 ***	0.740 ***		0.825 ***	0.697 ***		0.948 ***
	(0.037)		(0.041)	(0.029)		(0.039)	(0.044)		(0.049)	(0.029)		(0.038)	(0.039)		(0.041)
Spatial error term		0.812 ***	−0.255 ***		0.960 ***	0.239 **		0.718 ***	−0.284 **		0.947 ***	0.127 ***		0.788 ***	−0.346 ***
		(0.036)	(0.094)		(0.013)	(0.080)		(0.046)	(0.094)		(0.016)	(0.084)		(0.038)	(0.094)
Measures of fit															
Log likelihood	322.999	299.697	349.978	360.402	339.2448	386.346	365.005	350.530	389.438	350.695	330.045	378.271	287.152	271.201	317.570
AIC	−623.999	−579.393	−677.955	−698.804	−658.49	−750.692	−708.011	−681.061	−756.877	−679.39	−640.091	−734.542	−552.304	−522.402	−613.141
SC	−582.377	−541.555	−636.333	−657.182	−620.651	−709.07	−666.389	−643.222	−715.255	−637.768	−602.253	−692.920	−510.682	−484.563	−571.518
*R* ^2^	0.798	0.781	0.810	0.892	0.898	0.896	0.689	0.671	0.710	0.880	0.884	0.884	0.738	0.724	0.760
*N*	325	325	325	325	325	325	325	325	325	325	325	325	325	325	325

Note: *** *p* ≤ 0.001, ** *p* ≤ 0.01, and * *p* ≤ 0.05.
